# Molecular Mechanisms of Poxvirus Evolution

**DOI:** 10.1128/mbio.01526-22

**Published:** 2022-12-14

**Authors:** Greg Brennan, Ana M. M. Stoian, Huibin Yu, M. Julhasur Rahman, Shefali Banerjee, Jeannine N. Stroup, Chorong Park, Loubna Tazi, Stefan Rothenburg

**Affiliations:** a Department of Medical Microbiology and Immunology, School of Medicine, University of California, Davis, Davis, California, USA; Albert Einstein College of Medicine

**Keywords:** DNA recombination, evolution, gene duplication, horizontal gene transfer, poxviruses

## Abstract

Poxviruses are often thought to evolve relatively slowly because they are double-stranded DNA pathogens with proofreading polymerases. However, poxviruses have highly adaptable genomes and can undergo relatively rapid genotypic and phenotypic change, as illustrated by the recent increase in human-to-human transmission of monkeypox virus. Advances in deep sequencing technologies have demonstrated standing nucleotide variation in poxvirus populations, which has been underappreciated. There is also an emerging understanding of the role genomic architectural changes play in shaping poxvirus evolution. These mechanisms include homologous and nonhomologous recombination, gene duplications, gene loss, and the acquisition of new genes through horizontal gene transfer. In this review, we discuss these evolutionary mechanisms and their potential roles for adaption to novel host species and modulating virulence.

## INTRODUCTION

Poxviruses are a diverse family of viruses that can have significant impacts on both human and animal health. Within the *Chordopoxvirinae* subfamily, which infects vertebrates, 18 genera are currently recognized. Unlike many other viruses, poxviruses enter host cells by binding to receptors that are highly conserved between different species, such as glycosaminoglycans (1). Therefore, the poxvirus host range is independent of species-specific receptors, and productive infection is largely determined by how well they can antagonize the host immune response (2). Within the poxvirus family, there is significant phenotypic and genotypic variation between, and within, the different genera. For example, all Old World orthopoxviruses share 109 core genes, but the total gene complement ranges from up to 214 intact genes in cowpox viruses to variola virus, which encodes 162 intact genes and 17 truncated genes, the latter of which might still encode functional proteins (3, 4). Perhaps not surprisingly, this difference in gene content is also reflected in the viral host range since many of the “accessory” genes are involved in host range and immune evasion. Cowpox viruses typically have one of the broadest known host ranges among orthopoxviruses, whereas variola virus is only able to infect humans. Details of different types of poxviruses can be seen in Text Box 1.

TEXT BOX 1

**Orthopoxviruses**

**Variola virus** is the causative agent of human smallpox, which killed more than 500 million people in the last century alone. A WHO-led mass vaccination campaign, using the closely related vaccinia virus, eradicated variola virus from nature in 1977. Variola virus infection was restricted to humans, and mortality rates ranged from less than 1% to 30%. Changes in virulence were observed in multiple lineages over time (5).

**Vaccinia virus** is the most extensively studied poxvirus. Vaccinia virus has a broad host range, although the origin of the virus remains unclear. Both laboratory-adapted and feral strains exist that have many phenotypic and genotypic differences, including virulence and tissue tropism (6).

**Monkeypox viruses** can be grouped into two major clades, clade 1 (Central African origin) and clade 2 (West African origin), which display stark differences in virulence, for example, fatality rates of approximately 10% or less than 1%, respectively (7, 8).

**Cowpox viruses** are a heterologous group of poxviruses which represent at least five distinct orthopoxvirus species. Collectively, they have the broadest host range and largest genomes among poxviruses (9, 10).

**Leporipoxviruses**

**Myxoma virus** is a lagomorph-restricted virus. Its natural hosts are Tapeti and brush rabbits in the Americas, in which it causes a self-limiting and rarely fatal infection. However, in European rabbits, this virus causes a systemic infection with a near 100% mortality rate. Because of this high mortality, myxoma virus has been used as a biological agent to control European rabbit populations in Australia and Europe. Inadvertently, these releases have also become one of the best-studied examples of virus-host evolution in the field. Soon after the release in the field, myxoma virus became partially attenuated, and European rabbits became partially resistant (11).

Because they are double-stranded DNA (dsDNA) viruses and have DNA polymerases with proofreading capabilities, poxviruses are often thought to evolve relatively slowly for viruses (12). Until recently, most poxvirus evolutionary analyses were based on a relatively small number of consensus sequences. However, with the advent of deep sequencing technologies, we can now assess the frequency of minor variants in poxvirus populations. Differences in host selective pressure, such as during host switches, or by direct genome editing through host enzymes like the APOBEC3 family, are also beginning to be recognized as contributors to this standing variation in viral populations (13). Moreover, recent work has also demonstrated that poxvirus evolution is shaped through architectural changes just as much as it is shaped through single nucleotide variants (SNVs). These differences in both natural history and molecular biology have the potential to differentially shape the selective pressures each virus is subjected to (14). This work discusses the currently known molecular mechanisms influencing poxvirus evolution, highlighting areas of future study.

## POINT MUTATIONS

Poxviruses have been assumed to have relatively low mutational rates due to the fact that their genetic material is replicated by DNA polymerases with proofreading abilities. This paradigm was seemingly supported by epidemiologically linked variola virus isolates that had no nucleotide changes over periods of up to a year between samples (5). Similarly, genome sequences from two Tanapox isolates separated by 50 years had only 35 single nucleotide differences (15). In contrast, a report has identified an unexpectedly higher rate of SNV accumulation in recent monkeypox virus isolates, 7 SNVs since the initial outbreak in March 2022 and a total of 50 SNVs since 2018 (13).

The majority of substitution rate studies for dsDNA viruses have been focused on herpesviruses (14). Both herpesviruses and poxviruses encode a type B family member DNA-dependent DNA polymerase, UL30 in the case of herpes simplex virus 1 (HSV-1) and E9 in the case of vaccinia virus, and an underlying assumption of these studies is that the respective viral polymerases will behave in similar ways. The HSV-1 substitution rate has been estimated between ~1 × 10^−7^ and 1 × 10^−8^ substitutions/site/year (16–19). For variola virus, a substitution rate of ~1 × 10^−6^ substitutions/site/year was calculated, an approximately 10- to 100-fold difference from HSV-1 (14, 20). In contrast, the myxoma virus substitution rate, after its release as a biological control agent against European rabbits, was calculated to be ~1 × 10^−5^ substitutions/site/year, approximately 10-fold higher than the calculated variola virus substitution rate (21). Although many of the myxoma virus field isolates were attenuated, no individual SNV or pair of SNVs has yet conferred attenuation in reverse-engineered field strains, indicating that multiple mutations may be responsible for the attenuated phenotype, possibly through epistatic interactions (22).

The substitution rates for poxviruses suggest that, at least in some cases, the assumption that herpesvirus mutation rates approximate poxvirus mutation rates may not hold. One explanation for the difference in the substitution rates among poxviruses may be that variola virus was well adapted to the human population, whereas myxoma virus has been undergoing a recent host switch and was thus presumably subject to increased selective pressures. Host adaptation may also be an explanation for the higher-than-expected SNV accumulation in the recent monkeypox virus outbreak (13). In addition to the substitution rate itself, both the effective population size and the selection coefficient influence the probability that a new mutation will be fixed (23–25). Thus, one possible explanation for the difference between the epidemiological isolates and the phylogenetic observations is that selective sweeps or severe bottlenecks, either within a patient or during transmission between patients, may act to reduce accrued variability in an individual.

Recent experimental evolution studies in poxviruses provide support for this hypothesis, as multiple polymorphisms have emerged during serial passage in several studies (26–29). While these experimental evolution studies were not designed to elucidate the mutation rate of poxviruses, they underscore the fact that poxvirus populations are not monolithic but have standing variations. This population-level variation can, in combination with other mechanisms discussed in this review, allow poxviruses to respond rapidly to selective pressures.

## GENOME RECOMBINATION

**FIG 1:**
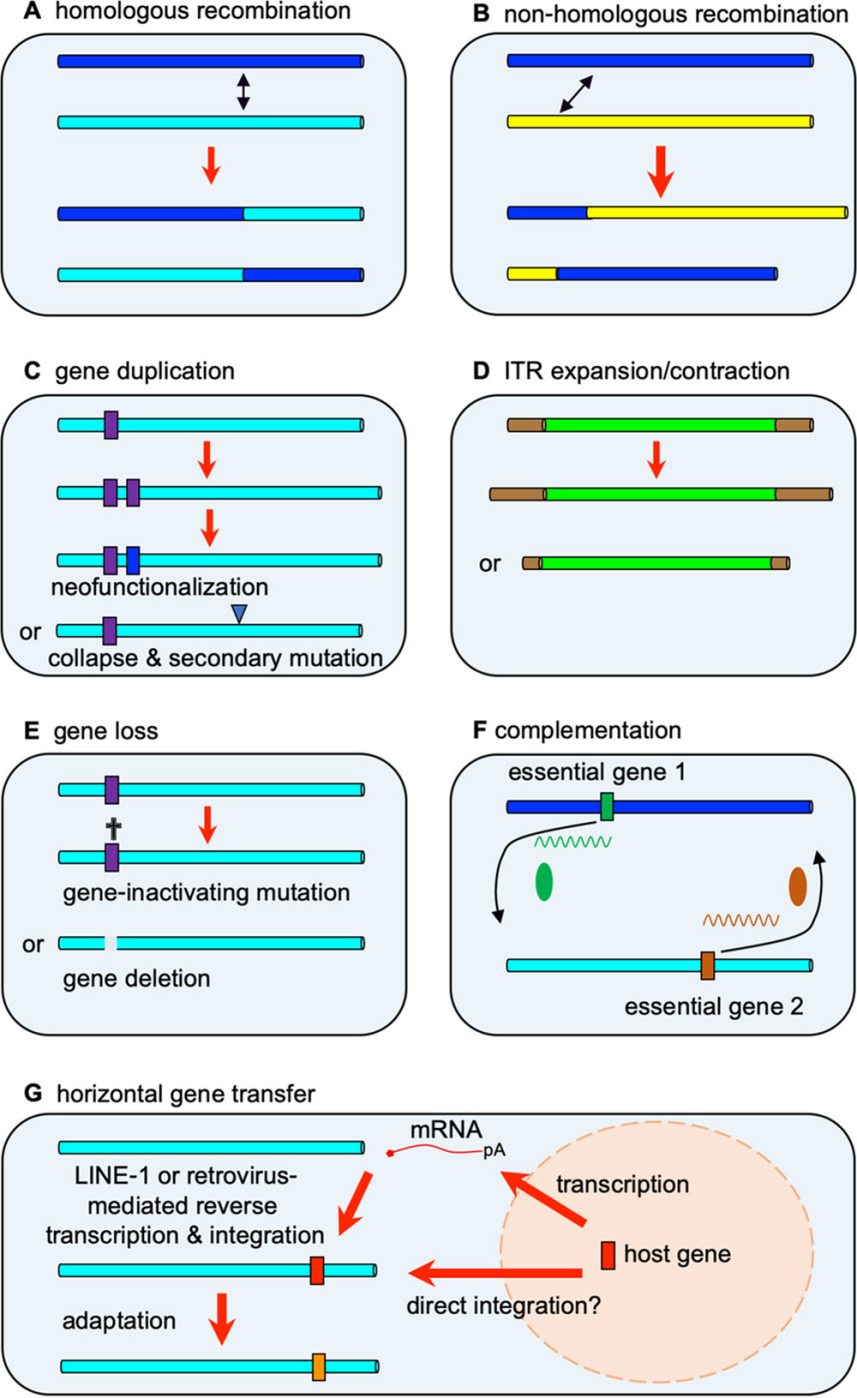
Mechanisms of poxvirus evolution. (A, B) Recombination can occur between homologous (A) or nonhomologous (B) sequences, either in *trans* between different genomes as shown here or in *cis* within a genome (not shown). (C) Duplications of genome sequences can lead to partial or complete gene duplications. Duplicated copies can acquire mutations that can lead to gene neofunctionalization, or increased gene dosage can lead to replication benefits that can increase the chance of beneficial secondary mutations, which can be followed by the collapse of the duplication. (D) ITRs can expand or decrease in size, which can result in duplicated genes in the ITR. (E) Gene loss can occur through gene-inactivating mutations and deletions. (F) Replication-incompetent viruses can be complemented by another virus. (G) Poxviruses can theoretically acquire host genes through DNA-dependent or RNA-dependent mechanisms, followed by adaptation through other mechanisms.

Poxviruses are highly recombinogenic, and recombination between different poxvirus species, strains, or isolates is a major driver for poxvirus evolution and genetic diversification (30). Recombination can be broadly grouped into homologous recombination and nonhomologous recombination, which can occur in *cis* within a genome or in *trans* between different genomes (Fig. 1A and B). In homologous recombination, recombination occurs between sequences that share sequence identity. In poxviruses, homologous recombination occurs at a high frequency, which has been exploited in the laboratory to readily generate recombinant poxviruses (31). In nonhomologous recombination, sequences with little or no sequence identity recombine, which is much rarer in poxviruses (32). The molecular mechanisms underlying poxvirus recombination are thought to involve multiple vaccinia virus proteins, particularly those involved in DNA repair processes. These mechanisms have been reviewed in detail recently and are therefore not extensively discussed in this review (33).

Recombination between different poxvirus strains or species was first described experimentally by detecting *in vitro* differences in plaque characteristics, temperature sensitivity of virus replication, and later by identifying changes in virulence in animal models (34–39). In experimental coinfections with closely related viruses, either myxoma virus and rabbit fibroma virus (Shope fibroma virus), or two different vaccinia virus strains, recombination has been detected at 1 event per 8 kb or 12 kb, respectively (28, 40). Recombination can also occur between regions within the same virus genome. Because these sequences are spatially tethered, this recombination can happen much faster than recombination between coinfecting viruses. During coinfection, the genomes are physically separated in distinct virus factories; therefore, recombination can only occur after these structures collide and fuse, about 5 to 6 h postinfection (28, 41).

There is strong evidence that recombination also occurs in natural poxvirus populations. A recent study identified extensive recombination between multiple cowpox virus clades, which represent at least five different orthopoxvirus species. This work demonstrates recombination both within the same virus species and also between more distant species (9). Providing more evidence for interspecies recombination, phylogenetic analysis of a cowpox virus isolated from a human demonstrated recombination not only with other cowpox virus clades but also with ectromelia virus, vaccinia virus, and the more distantly related Alaskapox virus (42). While the biological consequences of these cowpox virus recombination events are currently unclear, a natural recombination event involving myxoma virus contributed to a recent host switch, demonstrating that natural recombination events can have profound phenotypic impacts. A novel myxoma virus strain (myxoma virus Toledo) was recently isolated from wild Iberian hares that exhibited myxomatosis-like lesions. Genome analysis of the isolate revealed a 2.8-kb region carrying four genes (*M157*, *M158*, *M159*, and *M160*) with relatively low sequence identities to the myxoma virus genes *M060R*, *M061R*, *M064R*, and *M065R* (43). A subsequent study showed that the myxoma virus Toledo M159 protein, an ortholog of the vaccinia virus C7L family of host range factors, was essential for productive replication of the recombinant strain in hare and human cells (44). Overall, the data suggest that a recombination event between myxoma virus and an unidentified poxvirus facilitated a host species jump of the myxoma virus Toledo strain from rabbits to hares. In addition to host switching, recombination between the two virulent wild-type myxoma virus Lausanne and MSD strains unexpectedly resulted in an attenuated vaccine strain, SG33 (45, 46).

Recombination can also occur between naturally circulating poxviruses and poxvirus vaccine strains. For lumpy skin disease virus (LSDV), which affects cattle in large parts of the world (47), a recombinant virus was discovered in Russia that contained a mosaic LSDV genome combining sequences from a wild-type field strain and a vaccine strain. In this hybrid LSDV isolate, 27 recombination events were discovered with an average distance of 2.4 kb between recombination sites (48). This recombinant virus was repeatedly detected in subsequent LSDV outbreaks (49, 50). The phenotypic effects of these wild-type vaccine recombinants are not yet clear. However, based on data from experimental systems and naturally occurring viruses, recombination between naturally occurring poxviruses or vaccine strains has the potential to yield viruses with novel properties such as altered host range, transmissibility, or even modified immune evasion and virulence. Therefore, intense investigation and surveillance are required to identify potential recombinant viruses, especially during active poxvirus outbreaks.

## GENE DUPLICATIONS

Gene amplification has been a common theme for adaptation across all domains of life, including viruses (51, 52). In the presence of replication-limiting conditions, this can provide a rapid way to overcome selective pressure by increasing the copy number of one or more genes. This increased gene dosage can improve virus replication, presumably through mass action effects (52–55). In poxviruses, gene amplifications tend to rapidly collapse if the selective pressure is removed, likely due to increased fitness costs necessary to maintain amplification and the recombinogenic nature of poxviruses discussed above. The process of gene amplification and collapse has been termed “genomic accordions” (26). However, if the selective pressure persists, gene amplifications can facilitate mutation, neofunctionalization, or subfunctionalization (Fig. 1C).

Because gene amplifications are often transient events, it is difficult to observe viruses undergoing gene amplification in nature. However, there is substantial phylogenetic evidence for gene amplification leading to the establishment of gene families in poxviruses (4, 56). In general, the founding member of a gene family likely would have been horizontally acquired from the host, discussed below in the “Horizontal gene transfer” section. Paralogs of these genes likely would have arisen through gene duplication to establish the various gene families. There are four main gene families in orthopoxviruses that account for approximately half of the accessory genes. These are the poxvirus immune evasion (PIE) domain proteins, approximately half of which also contain the tumor necrosis factor (TNF) receptor domain (57), the B-cell lymphoma 2 (Bcl-2) protein family (58), the poxvirus and zinc finger (POZ) family characterized by an N-terminal BTB domain and Kelch repeats (59, 60), and ankyrin (ANK) repeat proteins, most of which also contain an F-box variant PRANC domain (61, 62). A recent study suggested that these families were largely established through three waves of gene duplication (4).

Maybe the most striking example of gene duplications in poxviruses is represented by the ANK/PRANC domain-containing gene family, with 35 intact ANK/PRANC genes present in the canarypox virus genome (63). In mammalian poxviruses, multiple gene duplication events have occurred independently in different lineages. In some lineages, the duplicated genes are in close proximity and often in tandem, whereas in other lineages, they are not linked, suggesting that these duplications are evolutionarily older and have migrated in the genome through recombination events (56, 64). These duplications have resulted in a multitude of biological functions, often targeting components of the host innate immune response (61, 62).

There are also examples of lineage-specific duplication events, such as the leporipoxvirus-specific duplication of the C7L-family genes 062, 063, and 064, which likely originated from two duplication events in a leporipoxvirus ancestor (65). In myxoma virus, these genes have evolved to have distinct protein interaction partners after duplication (66–68). For example, only M062 has been shown to interact with sterile alpha motif domain-containing protein 9 (SAMD9) and thereby inhibit a cGAS-dependent interferon response (67, 69). A recent study identified a similar example of duplication in cetacean poxvirus in which the virus genome was predicted to encode two tandem copies of the E3L ortholog, a full-length copy (CePV-TA-20) and a truncated copy (CePV-TA-21), which lacks the amino-terminal Zα domain (70, 71). E3L homologs without the Zα domain, like CePV-TA-21, are also found in other chordopoxviruses such as myxoma and monkeypox viruses (56, 71). Taken together, these studies demonstrate that gene duplication events during poxvirus evolution have contributed to an increased repertoire of host range genes and influence the various host ranges of these viruses.

In addition to the phylogenetic evidence, there is strong experimental evidence that gene amplification is an early and potent adaptive mechanism in poxviruses. The first evidence of adaptive gene amplification was documented in a selection study where vaccinia virus developed resistance to hydroxyurea, an inhibitor of ribonucleotide reductase, by amplifying its ribonucleotide reductase gene (72). Since this initial report, several experimental evolution studies have modeled poxvirus adaptation to overcome PKR inhibition in different hosts. Poxviruses encode two PKR antagonists, called E3L and K3L in vaccinia virus, which have species-specific differences in their ability to inhibit PKR (73–75). In the initial study, the authors serially passaged a vaccinia virus lacking E3L in human cells. They demonstrated an early and rapid gene amplification of the weak PKR antagonist K3L that was sufficient to fully rescue virus replication in human cells, presumably through mass action effects (26). This initial amplification facilitated the emergence of an adaptive SNV in the amplified K3L gene, which rapidly increased in frequency in the population. Taken together, these observations suggest that increased copy numbers can also provide an increased chance for adaptive mutations to emerge in the amplified gene. Follow-up studies by the same group demonstrated that these gene arrays can accelerate selective sweeps of SNVs, at least in part through a process of gene conversion (76, 77).

In a similarly designed study, a vaccinia virus expressing a weak PKR antagonist derived from rhesus cytomegalovirus (RhTRS1) also underwent rapid amplification of the *rhtrs1* locus to overcome PKR activity in African green monkey (AGM) cells (27). Unlike the previous study, adaptive mutations emerged in two other vaccinia virus genes, A24R and A35R, rather than in the amplified *rhtrs1* gene. In addition, this *rhtrs1* amplification also provided a replication benefit in otherwise completely resistant human- and rhesus macaque-derived cells compared to the parent virus. In a follow-up study, the authors demonstrated that the initial gene amplification acquired in the AGM cells was necessary for further adaptation to human cells (78). This adaptation was facilitated by another increase in *rhtrs1* copy number to overcome a more stringent barrier to replication in human cells, mediated by both PKR and RNase L. This suggests that gene amplification can overcome species-specific restriction barriers in different hosts through relatively nonspecific gene dosage effects and thus may act as a “molecular foothold” to facilitate viral spread to otherwise nonpermissive species (Fig. 1C).

In addition to facilitating the emergence of adaptive SNVs, gene duplication can result in neofunctionalization of existing genes. A recent study identified a rifampin-resistant vaccinia virus isolate carrying a duplication of a gene segment, which resulted in partial duplication of the A17 gene (79). The partially duplicated gene encoded a C-terminal-deleted A17 variant which, together with wild-type A17L, bound to the vaccinia virus scaffolding protein D13 and prevented its interaction with rifampin. This study provided the first evidence of an alternate mechanism of rifampin resistance, which previously was always associated with mutations in the D13 gene (80, 81). Furthermore, this work demonstrates that gene amplification in poxviruses can result in new gene functions by promoting neofunctionalization or subfunctionalization of existing genes. However, the truncation also had a dominant negative effect on replication fitness, and amplifications of A17L, including truncated copies, were lost immediately in the absence of rifampin.

Overall, these observations suggest that gene amplification provides a rapid mode of adaptation to different selective pressures by increasing gene dosage and providing, in some instances, a relatively nonspecific replication benefit. The amplification further increases the apparent rate of adaptation by expanding the number of gene copies that can acquire an adaptive mutation, as seen in the K3L study (26). Alternatively, gene amplification may also allow a virus population to sample adaptive mutations at different gene loci as demonstrated in the *rhtrs1* study, where the adaptive mutations were found in genes outside the amplified locus (27, 78). However, gene amplification balances these benefits with fitness costs, most notably in the requirement to replicate and support potentially very large gene arrays. This fitness cost is evident by the rapid contraction or loss of these amplified loci in the absence of selective pressure. The underlying mechanisms giving rise to the initial duplication event are poorly understood. However, once established, this duplication can be rapidly expanded or collapsed to a single copy by homologous recombination, as discussed above. Because of the relative rapidity with which duplications have emerged in experimental systems, preexisting gene duplications may be present at low frequencies in a virus population, although this has not yet been confirmed. Similarly, it is not yet known whether there are “hot spots” for gene duplication events or if they can occur across the viral genome.

## INVERTED TERMINAL REPEAT EXPANSIONS AND CONTRACTIONS

Poxviruses contain inverted terminal repeats (ITRs) near the ends of their genomes, which represent identical and oppositely oriented sequences. The length of the ITR varies between viruses, from less than 1 kb to more than 17 kb in length. Most poxviruses encode multiple genes in their ITRs, many of which are involved in immune evasion. Because these ITR-encoded genes are essentially duplicated, these gene products are generally expressed to a higher level through gene dosage effects. This relatively high expression, particularly of immunomodulatory genes, may play a role in the extensive host range of multiple poxviruses. For example, the human-restricted variola virus encodes relatively short ITRs, generally less than 1 kb, that do not contain any genes (5, 82). Mutations or gene insertions in one ITR region are usually also reflected in the other ITR region, probably through recombination (83–85).

The ITR also has the ability to expand or contract (Fig. 1D), and while the detailed mechanisms for these phenomena are currently unknown, they may involve nonhomologous recombination (86). One example is found in myxoma viruses: in the South American myxoma virus Lausanne and SLS strains, the ITR terminates within the open reading frame (ORF) of the K3L ortholog M156R, whereas in the related California MSW strain, the ITR extends the full length of M156R and encompasses three additional full-length genes. Consequently, these three genes, which all encode immunomodulatory proteins, are duplicated in the California MSW strain but not the Lausanne or SLS strains (46). A more direct example of ITR expansion is found in myxoma viruses that were released in Australia. In comparison to the parental SLS strain, some Australian lineages have expanded their ITRs to include full-length M156R and M154R genes (87). Different ITR lengths have also been observed in monkeypox viruses, where the ITRs in clade 1 (Central Africa) and clade 2 (West Africa) isolates are approximately 6.5 kb and 8.5 kb in length and contain four or six genes, respectively (7, 88). However, in one clade 1 isolate from Sudan, the ITR was 17.5 kb in length and contained 14 complete ORFs (89). Thus, ITR expansion represents a second mechanism to increase the gene dosage of select genes that is likely distinct from the gene duplication mechanism discussed above.

## GENE LOSS/GENE INACTIVATION

While gene gain is most frequently observed in poxvirus lineages that lead to distinct genera, gene loss or gene inactivation is a predominant motif during speciation within genera (3, 90, 91). Within the *Orthopoxvirus* genus, gene content correlates with host range, i.e., the genome of host-restricted variola virus contains 162 intact genes, whereas cowpox viruses, which have the largest host range, contain as many as 214 intact genes. As mentioned in the introduction, cowpox viruses possess the largest gene content among orthopoxviruses; however, no known cowpox virus isolate contain all the genes carried by the orthopoxvirus genus as a whole (4). In more host-restricted orthopoxviruses, gene loss often preferentially impacts host range or virulence genes (4, 56).

Gene loss/inactivation can occur through large deletions, smaller insertions and deletions (indels), SNVs that introduce premature stop codons, mutations in gene regulatory regions, and nonsynonymous SNVs introducing loss-of-function mutations (Fig. 1E). Microsatellites, which constitute about a quarter of the poxvirus genome, are a major source of new early stop mutations (92). The short-nucleotide motifs, between one and six nucleotides in length, that make up microsatellites are hot spots for evolution due to DNA slippage during replication and recombination (93).

One striking example of gene loss is found in orthologs of the vaccinia virus host range gene K1L. This gene is inactivated in the closely related variola virus, camelpox virus and taterapox virus. However, the gene-inactivating mutations are different in each virus and introduced unique stop codons or out-of-frame indels, suggesting that these genes were independently inactivated (56). Supporting this conclusion, two of four variola virus isolates from the medieval period have intact K1L orthologs, whereas the other two have unique gene-inactivating mutations (94). This shows that, even within a single lineage, multiple inactivating mutations can occur independently, and it indicates the importance of gene loss in poxvirus species radiation. Modern variola virus isolates also have a 17-bp deletion upstream of the K1L open reading frame that is predicted to disrupt the likely promoter motif. This region is intact in the four previously discussed ancient variola virus isolates, suggesting prolonged and independent selection for K1L inactivation in humans (56, 94). There are also 13 other genes that are inactivated in modern variola virus isolates but intact in at least 1 ancient variola virus isolate (94). Many of these genes also encode proteins with immunomodulatory functions.

In some cases, disrupted genes can maintain some of their function rather than being completely inactivated. For example, the vaccinia virus host range and virulence factor E3L encodes a protein containing an N-terminal Zα domain, which is important for inhibiting ZBP1-dependent necroptosis, and a C-terminal dsRNA binding domain, which acts as an inhibitor of PKR, and other dsRNA binding proteins (95, 96). As we discussed above, the Zα domain-encoding gene fragment is deleted or inactivated in some E3L orthologs, for example, leporipoxviruses, monkeypox virus, and volepox virus. In leporipoxviruses, the Zα-encoding portion of the gene is deleted, yet the protein product lacking the Zα domain is still able to inhibit PKR (97). In the monkeypox virus genome, the Zα-encoding part is present; however, the canonical E3L start codon is mutated, and two additional small deletions disrupt the ORF (56). However, a second start codon downstream within the Zα domain is utilized to also produce a protein product that lacks a functional Zα domain but retains dsRNA binding properties, which can inhibit PKR (98).

Protein loss of function can also be precipitated by SNVs or indels that do not disrupt the open reading frame. One such example is found in the myxoma virus K3L ortholog M156R, in which a Leu71Pro (previously known as Leu98Pro) mutation was identified in about half of the myxoma virus isolates from Australia (99). Even though the wild-type and mutated proteins were expressed to similar levels, the Leu71Pro mutation prevented PKR inhibition and attenuated the virus in cell culture. Thus, the ~50% of Australian isolates containing this mutation may promote extended survival of the European rabbit host relative to wild-type myxoma virus infection, which could lead to better transmission (100). It is important to note that these nondisruptive loss-of-function mutations are hard to predict and must be determined experimentally.

One explanation for the prevalence of gene loss in poxviruses may be that the large complement of immunomodulatory and host range accessory genes found in the poxvirus family may render some of these genes redundant. In these cases, redundant genes may be dispensable for infection of their host species, and thus, there may be little selective pressure to maintain some genes. Consequently, gene loss might indirectly select for a more limited host range, as these more broadly acting genes are inactivated over evolutionary time. Smaller genomes might provide advantages during replication, especially within a virus population. Alternately, or additionally, there is some evidence that gene loss may paradoxically compensate for inactivated genes in some circumstances. In one study using an attenuated vaccinia virus strain with a deletion in the B1R gene, the authors found compensatory inactivating mutations in the B12R gene, which increased virus replication (101). Similarly, inactivating mutations in A26L, G6R, and A14.5L increased viral fitness after serial passaging an attenuated vaccinia virus that contained the myxoma virus ortholog of the transcription factor A8R instead of the vaccinia virus gene (102). These reports indicate that, in some cases, gene inactivations can compensate for other attenuating mutations. Gene inactivation can also lead to increased virulence, as shown in a mouse model using a vaccinia virus strain lacking the B15R gene, which encodes a homolog of the interleukin-1β receptor (103).

## HORIZONTAL GENE TRANSFER

Horizontal gene transfer (HGT) is the exchange of genetic material between different organisms by asexual processes, and it can also occur between viruses and their hosts (104–106). A considerable number of predicted proteins in multiple dsDNA virus families share moderate to high sequence identities with host proteins, suggesting that their encoding genes have been captured from previous hosts via HGT (107–109). In poxviruses, putative horizontally acquired genes are distributed throughout the viral genomes and include some core genes that are found in most poxviruses (91, 110). Many of these horizontally acquired genes contribute to poxvirus fitness by counteracting the host immune system, extending the virus host range, and protecting from environmental damage, including interleukin-10, interferon gamma receptor, tumor necrosis factor (TNF) receptor, serpin, glutathione peroxidase, and deoxyribodipyrimidine photolyase homologs (91, 108, 110–112). In general, these viral genes are significantly shorter than their host counterparts, suggesting that there may be selective pressure, such as constrained genome size, to reduce horizontally acquired genes to the minimal functional domains of their more complex host counterparts (113).

Computational analysis suggests that a large proportion of poxvirus genes were horizontally acquired from their hosts (91). The majority of horizontal gene transfer events likely occurred in three distinct waves throughout poxvirus evolution (4). There are two broad mechanisms by which poxviruses might acquire host genetic material, either directly through DNA-mediated mechanisms, including integration via recombination or DNA transposons, or indirectly, including RNA-mediated mechanisms such as retrotransposons or retroviruses (Fig. 1G) (32, 114). Most of the poxvirus genes predicted to have been captured from hosts lack definitive genomic signatures of either mechanism. However, RNA-mediated mechanisms for HGT are supported by several phylogenetic lines of evidence. For example, the fowlpox virus genome contains a reticuloendotheliosis provirus, an avian retrovirus (115). In taterapox virus, there is a host-derived short interspersed nuclear element flanked by a perfect 16-bp target site duplication, a signature of long interspersed nuclear element-1 (LINE-1) retrotransposons (114). Furthermore, some orthopoxviruses encode homologs of the Golgi anti-apoptotic protein (GAAP), which shows about 76% protein identity with mammalian homologs, indicating relatively recent HGT (116). These viral genes are flanked by adjacent target site duplications and a putative poly(A) tail remnant, implicating LINE-1 in the capture of a GAAP host gene (84, 85).

Two recent experimental studies address the mechanism(s) of HGT in poxviruses (84, 85). In each study, the authors used a replication-deficient vaccinia virus, which lacks PKR inhibitors E3L and K3L and can only replicate in PKR-deficient cells or in the presence of PKR inhibitors provided in *trans*. To track host gene transfer, cell lines were stably transfected with plasmids encoding either E3L or K3L preceded by an intron. These stably transfected cells were infected with the replication-deficient viruses, and the progeny virions were then used to infect PKR-competent cells. This selection strategy only allows replication of viruses that took up a PKR inhibitor from the initial cell line. In these studies, a combined 30 HGT events were identified. In all cases, the introns were spliced out, and poly(A) tails were present. Twenty-six of the transferred genes also contained target site duplications with an average length of 16.2 bp. These genetic signatures are characteristic of LINE-1-mediated transposition of host RNA into the viral genome. In one study, the captured genes primarily integrated outside the central region (84), while in the other study, captured genes were distributed throughout the genome, including the central conserved region (85). In some cases, integration was found in essential genes, which presumably inactivated these genes. In those cases, the derived virus was rescued by coinfection with the parental virus, enabling the viruses to complement each other (Fig. 1F). After serial passaging, the viruses formed a replication-competent virus through a process of recombination to generate tandem arrays that contained both the uninterrupted essential gene and the essential gene disrupted by the horizontally acquired PKR inhibitor (85). Importantly, this study demonstrated that rescuing these essential gene disruptions required a cascade of events linking different evolutionary mechanisms to generate replication-competent viruses. The approximate gene transfer rates in these experiments were between 1 in 23 to 50 million viable virions (84, 85). These calculations likely underestimate the actual transfer frequency because integrations into essential genes require the presence of a complementing virus. Taken together, these studies demonstrate that the HGT of a particular gene is a very rare process. However, because the genes used to select for HGT represent only one of thousands of genes and transposable elements in the host cell, the actual HGT frequency is likely much higher but, in most cases, may not be maintained due to detrimental or neutral effects on virus fitness.

## CONCLUSIONS

Here, we have reviewed the many mechanisms underlying poxvirus evolution at both the nucleotide and architectural levels. There is evidence that the poxvirus substitution rate can, at least in some cases, be faster than in other dsDNA viruses. However, to the best of our knowledge, there have not yet been any studies rigorously defining the fidelity of the poxvirus DNA polymerase. In poxviruses, as with other organisms, the vast majority of mutations probably do not provide selective advantages for the virus, and mutations therefore do not become fixed. However, evolutionary changes can have unpredictable effects on viral fitness in both closely and distantly related host species. Therefore, a small subset of mutations in a viral population, by chance, may provide a replication benefit in an otherwise restricted environment, such as a host change, and become fixed (117).

In addition to nucleotide changes, architectural changes play substantial roles in poxvirus evolution. These mechanisms can enhance virus replication themselves through mass action effects, altering extant gene structure to generate new functions, or acquiring new genetic material from coinfecting viruses or the host. In addition, these mechanisms can and do work in concert. For example, the additional genes generated during duplication events can each independently acquire SNVs, which may increase the apparent rate of SNV accumulation without changing the underlying mutation rate. It is important to note that if these changes lead to better virus replication, the chance of acquiring additional mutations is increased both in the amplified gene itself, as well as generally throughout the genome because there are simply more genomes produced. Similarly, knockout of essential genes can be compensated for by a cascade of, first, coinfection complementing the virus in *trans*, followed by recombination to generate replication-competent virions with new genetic material and new functions (85). Gene inactivations in a lineage could also be reversed by recombination, which might explain why cowpox viruses, which show strong evidence of frequent recombination, show such a high gene content in all lineages. It is important to note that other mechanisms, in addition to the ones described here, may also cause architectural changes, such as gene fusion (4). Additionally, DNA polymerase slippage in microsatellites can cause in-frame indels, resulting in altered amino acid repeat numbers in proteins, as exemplified by a poly(aspartic acid) stretch of various lengths in the homologs of vaccinia virus A26 (OPG153) (92). Not unexpectedly, variations in microsatellites are also found in the current monkeypox virus outbreak, including in the A26 ortholog (118).

The concerted and sometimes transient nature of these evolutionary changes can be difficult to detect in nature; however, the combination of phylogenetic analysis and experimental evolution will continue to be a powerful method to unravel poxvirus evolution and identify evolutionary biomarkers of high-risk viruses. It is therefore important to be aware of the role of architectural changes in poxvirus evolution and ensure that bioinformatic pipelines used for poxvirus sequencing are able to detect these changes. Using whole-genome sequencing pipelines is even more important during emerging outbreaks because these architectural changes are generally not detected by standard screening procedures, and multiple lines of evidence demonstrate that they can arise rapidly in a population and cause substantial phenotypic changes.
